# Built Environmental Correlates of Walking and Cycling in Dutch Urban Children: Results from the SPACE Study

**DOI:** 10.3390/ijerph7052309

**Published:** 2010-05-06

**Authors:** Sanne I. de Vries, Marijke Hopman-Rock, Ingrid Bakker, Remy A. Hirasing, Willem van Mechelen

**Affiliations:** 1 Department of Health Promotion, TNO Quality of Life, Wassenaarseweg 56, 2301 CE Leiden, The Netherlands; E-Mails: marijke.hopman@tno.nl (M.H.-R.); ingrid.bakker@tno.nl (I.B.);; 2 Body@Work TNO VUmc,, EMGO Institute for Health and Care Research, VU University Medical Center, Van der Boechorststraat 7, 1081 BT Amsterdam, The Netherlands; 3 Department of Public & Occupational Health, EMGO Institute for Health and Care Research, VU University Medical Center, Van der Boechorststraat 7, 1081 BT Amsterdam, The Netherlands; E-Mails: ra.hirasing@vumc.nl (R.A.H.); w.vanmechelen@vumc.nl (W.v.M.)

**Keywords:** walking, bicycling, physical activity, urban design, infrastructure, child, linear regression

## Abstract

This study examined built environmental correlates of children’s walking and cycling behavior. Four hundred and forty-eight children from 10 Dutch neighborhoods completed a seven-day physical activity diary in which the number of walking and cycling trips for transportation, to school, and for recreation were assessed. The associations between observed built environmental characteristics and children’s walking and cycling behavior were examined with multivariate linear regression analyses. The results showed that built environmental correlates of children’s walking and cycling behavior differ by purpose and by commuting mode implying a behavior-specific approach for interventions and for future, preferably prospective, studies.

## 1. Introduction

Trend studies suggest a worldwide decline in children’s physical activity level, including active transportation [1]. In the last decades, an increasing proportion of children are being transported to school by car [2–4]. Although walking and cycling to school are associated with an increased level of overall active transportation, physical activity, energy expenditure, and cardiovascular fitness [5,6], active transportation to school has long been an overlooked source of daily physical activity for children [7]. However, during the past five years it has become one of the main components of strategies to increase children’s overall physical activity level [1].

In order to develop effective strategies that promote active transportation among children, determinants need to be understood. Social ecological models are increasingly being used to gain an insight in the role of the built environment in walking and cycling [8]. In studies using these models, active transportation has been found to be associated with street connectivity [9–11], land use mix [12], distance to a destination [12,13], steep inclines [11], neighborhood safety (e.g., windows facing the street) [11,14–16], traffic safety (e.g., heavy traffic, traffic lights, pedestrian crossings, and limited public transport) [9–11,15,17–19], neighborhood aesthetics [14,18], presence of street trees [12], and facilities near home (e.g., walking and cycling trails, stores, parks, and sports fields) [13]. Most of these studies have been performed in the United States and Australia [13,21]. Built environmental correlates found in these continents cannot easily be generalized to the Netherlands or other European countries [13,22,23]. The Netherlands has a different land-use pattern, geared to the needs of pedestrians and cyclists. Most Dutch children live within five kilometers from elementary school [24,25] and walk or cycle to it [26]. In countries in which distance is not a barrier for active transportation [9], other built environmental characteristics may be important, such as the presence of green space and water in the neighborhood, the absence of garbage and dog waste, and the walking and cycling infrastructure (e.g., cycle-tracks, zebra crossings, roundabouts, parallel parking spaces, and 30-km speed zones) [22].

Built environmental characteristics that correlate with children’s walking and cycling behavior may not only be ‘country or continent specific’, but also ‘purpose specific’. For example, studies among adolescents and adults have shown different built environmental correlates of walking and cycling for transportation than that of walking and cycling for recreation [27–29]. Among children, few environmental studies have focused on active transportation to other destinations than school or on recreational walking and cycling [5,13]. In addition, correlates of walking and cycling may also be ‘behavior specific’, thus built environmental correlates of walking may be different from those of cycling [23,30]. To our knowledge no studies exist that have examined the association between built environmental characteristics and children’s walking and cycling behavior separately for walking and for cycling. Therefore, this study aimed to identify built environmental correlates of (a) walking and cycling for transportation, (b) walking and cycling to school, and (c) walking and cycling for recreation among a sample of Dutch children. Furthermore, it was examined whether built environmental correlates of walking are different from those of cycling.

## 2. Methods

### 2.1. Study Design and Study Population

This study is part of the Spatial Planning and Children’s Exercise (SPACE) study [22], a study in which cross-sectional data were collected among a convenience sample of six to 11-year-old children from 10 disadvantaged neighborhoods in six cities in the Netherlands. Table 1 shows some general characteristics of the 10 neighborhoods. Five of the 10 neighborhoods were selected from a list of 56 disadvantaged neighborhoods designated by the government for spatial restructuring. Besides, five neighborhoods were included to investigate the effects of changes in the built environment on children’s physical activity in the future. Neighborhood selection criteria were inclusion of pre- and post-World War II neighborhoods, variation in the type of residences and the amount of green space between the neighborhoods, and presence of at least two elementary schools per neighborhood. Neighborhood boundaries were defined by city councils. The 10 neighborhoods varied in size and in population density. The children who lived in these neighborhoods and who were between six and 11-years old were recruited from 20 elementary schools (two schools per neighborhood). Children in this age group were studied, because they are more likely to have a radius of action for their daily physical activities that corresponds with the size of their neighborhood than younger children who have a smaller radius of action (*i.e.*, one block of houses) or older children who have a larger radius of action (*i.e.*, several neighborhoods) [31]. Informed consent was obtained from the parents of 1,228 children. All measurements were conducted between October 2004 and January 2005. The study was approved by the ethics committee of the Leiden University Medical Center.

### 2.2. Dependent Variables: Walking and Cycling

Walking and cycling were assessed by a validated seven-day physical activity diary [32], which was completed by one of the parents together with their child. During seven consecutive days for all waking hours, all physical activities were noted at the end of each day, including the duration of the activity and one of the following corresponding physical activity categories: active transportation, activities during school time, organized sports, playing outdoors, and activities at home. For each day, the number of walking and cycling trips were summed, distinguishing between trips for transportation, trips to school (*i.e.*, walking and cycling for transportation between 07:00–09:00 AM on school days (one-way trip)) and trips for recreation (*i.e.*, walking and cycling during playing outdoors and walking the dog).

### 2.3. Background Variables

Other variables that were collected at the individual level included: age, sex, body height and body weight of the child, maternal and paternal education level (low: primary school or lower vocational training; medium: secondary school or intermediate vocational training; high: higher vocational training or university), and country of origin of the child and both parents. Body height and body weight were measured by two trained research assistants with a portable stadiometer (Stanley 04–116) and a digital scale (Seca 812), respectively, while the child was wearing clothes but no shoes. Body mass index (BMI) was calculated (kg/m^2^) and categorized into normal weight, overweight, and obesity (excluding overweight) according to international age- and sex-specific cut-off points [33]. Children were categorized into Dutch, Turkish, Moroccan, Surinam/Antillean, other Western, and other non-Western ethnic groups. They were only categorized as Dutch if the child and both parents were born in the Netherlands.

### 2.4. Independent Variables: Built Environmental Characteristics

Built environmental characteristics were collected by neighborhood observation. Two trained research assistants walked through the neighborhoods after school time and together completed one checklist in unison. The checklist is based on the Neighborhood Environment Walkability Scale [34] but was modified to reflect the Dutch built environment, including items relevant to children (e.g., playgrounds, school yards, and dog waste), as identified by focus group interviews prior to the study [35]. The inter-rater reliability of the checklist was evaluated as good (percentage of agreement = 77%) in a later study [36]. The checklist consists of 54 items on type of residences, sports facilities, recreation facilities, play facilities, green space, water, dirt, traffic safety, walking and cycling infrastructure, and general impression of the activity-friendliness of the neighborhood [22]. Type of residences (8 items) were weighted and summed per neighborhood to estimate residential density [34]. The number of sports facilities (12 items), recreation facilities (3 items), and play facilities (4 items), and the scores on the presence of dirt (2 items) and traffic safety (5 items) were also summed per neighborhood. The remaining items were analyzed separately. These included: proportion of residents to enterprises, proportion of green space to residents, frequency of unoccupied houses (scoring: 1 [none]–5 [all]), presence of green space (scoring: 1 [no]–4 [much]), presence of water (scoring: 1 [no]–4 [much]), frequency of 14 items on walking and cycling infrastructure (*i.e.*, sidewalks, cycle-tracks, zebra crossings, pedestrian crossings, traffic lights, traffic islands, parking garages, parallel parking spaces, parking lots, speed bumps, 30-km speed zones, low-traffic zones, roundabouts, and intersections; scoring: 1 [few]–3 [many]), and general impression of the activity-friendliness of the neighborhood (scoring: 1–10).

### 2.5. Statistical Analyses

Fifty-one percent (n = 625) of the original sample returned the physical activity diary. Children failing to complete the diary for at least three school days (n = 68) and children with missing data on age, sex, parental education level (n = 108), and/or ethnicity (n = 82) were excluded from analyses. The final sample consisted of 448 children. This sample was older than the original sample of 1,228 children (8.3 ± 1.5 year old *versus* 7.8 ± 1.5 year old; t = −5.585; df = 1,226; p < 0.001) and more often of Dutch origin (χ^2^ = 44.003; df = 1; p < 0.001). No significant differences were found in sex and parental education level between the final and original samples.

Univariate and multivariate linear regression analyses were conducted in SPSS 14.0 to examine the association between 23 of the 26 built environmental characteristics and the number of walking and cycling trips per week. The following three built environmental characteristics were excluded from analysis because of insufficient variance between neighborhoods: the frequency of parking garages, speed bumps, and low-traffic zones. Dependent variables were: walking and cycling for transportation (model 1), walking and cycling to school (model 2), and walking and cycling for recreation (model 3). Walking and cycling were examined jointly and separately. First, the association between children’s walking and cycling behavior and built environmental characteristics were examined in separate models since the environmental characteristics may be correlated. After crude analyses, all models were adjusted for age, sex, parental education level, and ethnicity [13]. Built environmental characteristics that reached significance (p ≤ 0.05) in the adjusted univariate linear regression analyses were included in the multivariate linear backward regression analyses. Regression coefficients (B) and 95% confidence intervals (CI) were calculated. Although clustering of subjects within neighborhoods was limited (*i.e.*, intra cluster correlation (ICC) = 0.02), multilevel analyses were also conducted.

## 3. Results and Discussion

### 3.1. Results

The final sample consisted of 216 boys and 232 girls with a mean age of 8.3 ± 1.5 years (Table 2). Twenty-eight percent of the children were overweight or obese. The majority of the children was of Dutch origin and had parents with a medium education level. All 10 neighborhoods were represented in the final sample. The number of participants per neighborhood ranged between two and 108, with an average of 45 children.

**Figure 1.**
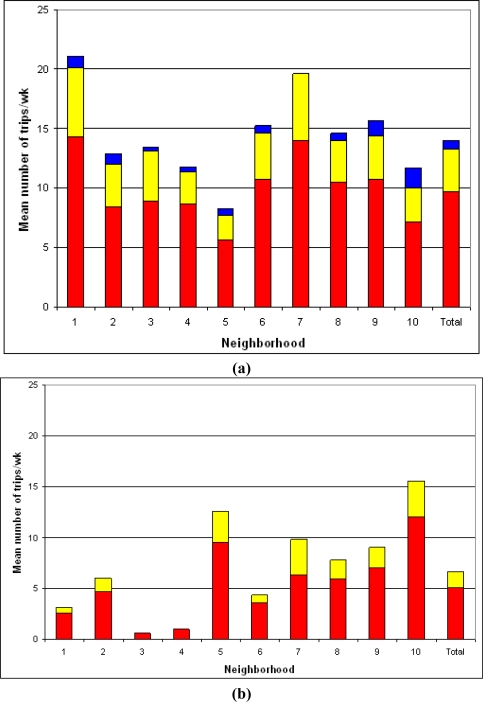
**(a)** Walking behavior per neighborhood: red = walking to school; yellow = walking for transportation (excluding to school); blue = walking for recreation. **(b)** Cycling behavior per neighborhood: red = cycling to school; yellow = cycling for transportation excluding to school.

Ninety-three percent of the children walked or cycled for transportation at least once per week. Walking or cycling to school was reported by 82% of the children. Of those children, 53% walked and 40% cycled to school on every school day. Twenty-five percent of the children walked for recreation at least once per week. Cycling for recreation was not reported. On average, the children made 13.3 ± 10.7 walking and 6.6 ± 9.2 cycling trips per week for transportation; 3.6 ± 3.2 walking and 1.5 ± 2.6 cycling trips per week to school; and 0.7 ± 2.0 walking trips per week for recreation. Significant differences were found between the neighborhoods in the use of walking and cycling for transportation, and walking and cycling to school (walking for transportation: range = 7.7–20.1 trips per week; F = 3.338; df = 9; p = 0.001, cycling for transportation: range = 0.6–12.6 trips per week; F = 8.970; df = 9; p < 0.001, walking to school: range = 2.1–5.8 trips per week; F = 3.346; df = 9; p = 0.001, cycling to school: range = 0.0–3.5 trips per week; F = 7.602; df = 9; p < 0.001) (Figure 1a and 1b). No significant differences were found between neighborhoods in the frequency of walking for recreation (range = 0.0–1.7 trips per week).

Univariate linear regression analyses showed that 15 of the 23 built environmental characteristics under study were significantly associated with walking or cycling for transportation or walking or cycling to school. These included: recreation and play facilities, green space, water, traffic safety, and items on the walking and cycling infrastructure. When these characteristics were entered in a multivariate model, total walking and cycling for transportation were strongest associated with the presence of water and the frequency of sidewalks in the neighborhood (R^2^ = 34.3%) (Table 3). Sidewalks remained significantly associated with total walking and cycling for transportation in a multilevel model (B = 7.41; 95% CI = 0.41–14.42; p < 0.05). However, when walking and cycling for transportation were analyzed separately, different built environmental correlates were found. Walking for transportation was strongest associated with the frequency of cycle-tracks, traffic lights, and roundabouts in the neighborhood in a multivariate model (R^2^ = 29.9%). For cycling for transportation, correlates were the number of recreation facilities, traffic safety, and the frequency of sidewalks, pedestrian crossings, traffic lights, parallel parking spaces, and parking lots in the neighborhood (R^2^ = 33.2%). In multilevel analyses, traffic lights and roundabouts remained associated with walking for transportation (B = −7.16; 95% CI = −17.85–−3.53; p < 0.10; B = 11.55; 95% CI = 1.49–24.51; p < 0.10, respectively). For cycling for transportation, significant correlates were the number of recreation facilities (B = 1.84; 95% CI = 0.05–3.64; p < 0.05) and the frequency of pedestrian crossings (B = 14.53; −3.91–32.96; p < 0.10) in a multilevel model. Comparable results were found for walking and cycling to school. Total walking and cycling to school were strongest associated with the frequency of cycle-tracks in the neighborhood in a multivariate model (R^2^ = 33.4%), whereas walking to school was strongest associated with the presence of green space and the frequency of pedestrian crossings, parallel parking spaces, parking lots, and roundabouts in the neighborhood (R^2^ = 30.0%), and cycling to school was strongest associated with the number of recreation facilities, the presence of green space, and the frequency of pedestrian crossings, traffic lights, and parallel parking spaces in the neighborhood (R^2^ = 30.6%). The environmental correlations observed for the total of walking and cycling to school as well as those of walking to school all remained significantly associated in multilevel analyses (*i.e.*, frequency of cycle-tracks: B = 1.32; 95% CI = −0.05–2.69; p < 0.10; green space: B = −2.59; 95% CI = −3.67–−1.50; p < 0.01; pedestrian crossings: B = 6.54; 95% CI = 3.51–9.56; p < 0.01; parallel parking spaces: B = 2.80; 95% CI = 1.64–3.96; p < 0.01; parking lots: B = 2.75; 95% CI = 1.58–3.91; p < 0.01; roundabouts: B = 4.31; 95% CI = 2.67–5.96; p < 0.01). For cycling to school, only the number of recreation facilities remained significantly associated in a multilevel model (B = 0.40; 95% CI = 0.11–0.69; p < 0.05). None of the built environmental characteristics under study were significantly associated with walking for recreation.

### 3.2. Discussion

The purpose of this study was to identify built environmental characteristics that were associated with children’s walking and cycling behavior and to examine whether there are differences by purpose (transportation, school, and recreation) and by commuting mode (walking, cycling, and combined). Ninety-four percent of the children in this study walked or cycled at least once per week. Although this is a high percentage compared to other countries, only a small percentage of the children (less than 10%) walked or cycled every day. Changes in the built environment may increase children’s walking and cycling behavior [37]. In our study, a number of built environmental factors were found to correlate with children’s walking and cycling behavior. Although small differences were found between the built environmental correlates of walking and those of cycling, both commuting modes were positively associated with the frequency of pedestrian crossings and the frequency of parallel parking spaces in the neighborhood. More differences were found in built environmental correlates of walking and cycling for different purposes. Whereas none of the built environmental characteristics under study were significantly associated with walking for recreation, a considerable proportion of the variance in walking and cycling for transportation and walking and cycling to school could be explained by built environmental characteristics. Adjusted multivariate models showed that about 30% of the variance in walking and cycling for transportation could be explained by the number of recreation facilities in the neighborhood and the walking and cycling infrastructure of the neighborhood. Comparable correlations were found for walking and cycling to school.

The results of our study are in line with studies among adults and adolescents, in which behavior specific built environmental factors correlating with walking and cycling were also found [27–29]. Carver *et al.* [27] found differences between the built environmental factors correlating with cycling for recreation, cycling for transportation, and cycling to/from school, and of walking for exercise, walking for transportation, walking to school, and walking the dog among Australian adolescents. Walking for transportation was associated with local traffic volume and stores near home. Cycling for transportation on the other hand, was associated with having good sports facilities. For walking and cycling to school, Carver *et al.* [27] found no significant associations, except for girls walking to school. For girls, parents’ concern about busy traffic was negatively associated with walking to school. From a review of environmental studies on walking among adults [28], it was concluded that built environmental characteristics associated with walking for exercise are different from those associated with walking for transportation. For cycling among adults, comparable conclusions can be drawn [29].

Although in our study, built environmental correlates of children’s walking and cycling behavior differed by purpose, there were also points of similarity. Both walking and cycling for transportation, walking and cycling to school, and walking for recreation were not associated with the residential density, type of residences, presence of dirt or the general impression of the activity friendliness of the neighborhood. Built environmental characteristics most consistently associated with walking and cycling for transportation and walking and cycling to school were the frequency of pedestrian crossings and parallel parking spaces in the neighborhood. In previous studies, the walking and cycling infrastructure of a neighborhood was also shown to be important [13,17,19]. For example, in the United States, Kerr *et al.* [20] found a positive association between perceived walking and cycling facilities (e.g., sidewalks, walking and cycling trails) within 1-km of the child’s residence and the objective walkability of the neighborhood. Hume *et al.* [19] found that adolescents whose parent perceived there to be insufficient traffic lights and pedestrian crossings in their neighborhood were less likely to increase their walking or cycling behavior to school in a two year period.

The positive association between the frequency of parallel parking spaces in the neighborhood and cycling for transportation, and walking and cycling to school in our study, might be explained by a lower speed of motorists in narrow streets [38]. In our study, the parallel parking spaces were frequently found in neighborhoods with 30-km speed zones (correlation coefficient r = 0.64; p < 0.001), sport fields (r = 0.62; p < 0.001), and less heavy lorry and bus traffic (r = −0.65; p < 0.001). Children may also feel safer when walking on the sidewalk; because of the barrier the parked cars create [35].

In this study, no associations were found between environmental characteristics of the neighborhood and walking for recreation. Perhaps walking for recreation takes place outside the boundaries of the neighborhood or perhaps other factors are important, such as children’s and parents’ perceptions of traffic safety, personal safety, and social interaction [19,20,27,39].

### 3.3. Limitations and Strengths

Our study had a cross-sectional design restricting the interpretation of directions of associations. However, in due course, measurements will be repeated after spatial restructuring of five of the 10 neighborhoods to examine the effects of environmental changes on children’s physical activity level. A second limitation is the limited variance in the built environment between the 10 neighborhoods. All neighborhoods were relatively deprived. Any future study should be extended to more rural neighborhoods to add variance.

This study is among the first to examine the association between the built environment and children’s walking and cycling behavior by purpose, and by commuting mode. Improving the walking and cycling infrastructure of neighborhoods (*i.e.*, building more pedestrian crossings and providing parallel parking spaces) may help to increase active transportation among children. However, one has to keep in mind that walkable neighborhoods are not necessarily cyclable neighborhoods (and *vice versa*), and neighborhoods which are very suitable for walking and cycling for transportation are not necessarily suitable for walking and cycling for recreation. Therefore, a behavior specific approach is recommended for interventions and future studies. Furthermore, future studies should explore the relative importance of, and the interaction between, individual, social environmental, built environmental, and natural environmental determinants of children’s walking and cycling behavior. Next, further research requires longitudinal and intervention studies, utilizing multilevel design methodologies. Since environmental characteristics are thought to have the potential to influence behavior without individuals being consciously aware of them, it is recommended to assess both perceived and objective characteristics of the built environment in the same study [40]. Objective measures of built environmental characteristics can be integrated in a geographic information system (GIS) [9,13,41,42]. In addition, the use of global position systems (GPS) can increase our understanding of children’s walking and cycling behavior, for example by providing an insight into walking and cycling routes to school [43,44]. Combining different subjective and objective measures of physical activity with different perceived and objective measures of the built environment will result in a picture of where, when, and how long children are exposed to certain built environmental characteristics and how these characteristics influence their walking and cycling behavior.

## 4. Conclusions

In this study, a broad array of potentially relevant characteristics of the Dutch built environment that may either encourage or discourage walking and cycling among children have been examined. The results showed that built environmental correlates of children’s walking and cycling behavior differ by purpose (transportation, school, and recreation) and by commuting mode (walking, cycling, and combined). In general, the higher the frequency of pedestrian crossings and the higher the frequency of parallel parking spaces in the neighborhood, the higher the number of walking and cycling trips for transportation purposes. In order to examine whether changing the traffic infrastructure of a neighborhood will actually lead to more walking and cycling among children, repeated measurements are required.

## Figures and Tables

**Table 1.** General characteristics of the 10 Dutch neighborhoods.

**Neighborhood**	**Main construction period**	**Size (hectare)**	**Population density (residents/km^2^)**
1. Delftwijk, Haarlem	post-WW II	51	8,646
2. Molenwijk, Haarlem	post-WW II	228	4,390
3. Spangen, Rotterdam	pre-WW II	65	16,278
4. Nieuwe Westen, Rotterdam	pre-WW II	124	15,076
5. Randenbroek-Schuilenburg, Amersfoort	post-WW II	215	5,511
6. Liendert, Amersfoort	post-WW II	105	7,234
7. Groenoord, Schiedam	post-WW II	110	7,955
8. Holy-Zuid, Vlaardingen	post-WW II	190	5,871
9. Berflo Es, Hengelo	pre-WW II	110	5,391
10. Wilderinkshoek-Tuindorp, Hengelo	pre-WW II	102	4,703

**Table 2.** Background characteristics of the sample (n = 448).

Sex	%

Boys	48
Girls	52

Age	year

Mean	8.3
Standard deviation	1.5

BMI (body mass index)	%

Normal weight	72
Overweight	20
Obesity	8

Origin	%

Dutch	61
Turkish	13
Moroccan	6
Surinam/Antillean	6
Other Western	6
Other non-Western	8

Maternal education	%

Low	27
Medium	56
High	17

Paternal education	%

Low	33
Medium	42
High	24

Neighborhoods	n

Delftwijk, Haarlem	26
Molenwijk, Haarlem	63
Spangen, Rotterdam	35
Nieuwe Westen, Rotterdam	31
Randenbroek-Schuilenburg, Amersfoort	46
Liendert, Amersfoort	67
Groenoord, Schiedam	2
Holy-Zuid, Vlaardingen	108
Berflo Es, Hengelo	60
Wilderinkshoek-Tuindorp, Hengelo	10

**Table 3.** Adjusted multivariate models of the association between built environmental characteristics and children’s walking and cycling behavior.

**Built environmental characteristic**	**Range**	**B 95% CI**					
		**Walking and cycling for transportation**_**1**_	Walking for transportation_1a_	Cycling for transportation_1b_	**Walking and cycling to school**_**2**_	Walking to school_2a_	Cycling to school_2b_
Play facilities	2–12						
Recreation facilities	0–11			1.66** 0.46, 2.86			0.41** 0.19, 0.63
Green space	2–3					−2.05** −3. **20, −0.91**	0.52 −0.27, 1.30
Proportion green space to residents	5–40						
Water	1–3	1.56 −2.71, 5.82					
Traffic safety	0–6			0.08 −1.11, 1.27			
Sidewalks	2–3	6.43* 1.32, 11.53		−2.14 −9.00, 4.71			
Cycle-tracks	1–2		3.45 −0.21, 7.12		1.12* 0.18, 2.05		
Pedestrian crossings	1–2			14.66** 5.96, 23.36		4.85** 1.65, 8.04	3.41** 1.49, 5.33
Traffic lights	1–2		−7.29** −10.71, −3.87	−1.385 −6.625, 3.855			−0.59 −1.61, 0.43
Traffic islands	1–2						
Parallel parking spaces	1–3			6.52* 1.18, 11.86		2.29** 1.08, 3.51	1.46** 0.38, 2.55
Parking lots	1–3					2.35** 1.13, 3.56	
Roundabouts	1–2		11.14** 6.35, 15.94			3.59** 1.80, 5.39	
Intersections	1–3						

B corresponds with the increase or decrease of the number of walking or cycling trips per week with an increase of one unit in the particular built environmental characteristics, independent of other characteristics in the model;

CI = confidence interval;

* p < 0.05;

** p < 0.01;

model 1: R^2^ = 34.3%;

model 1a: R^2^ = 29.9%;

model 1b: R^2^ = 33.2%;

model 2: R^2^ = 33.4%;

model 2a: R^2^ = 30.0%;

model 2b: R^2^ = 30.6%.
